# *N*-alkylisatin-based microtubule destabilizers bind to the colchicine site on tubulin and retain efficacy in drug resistant acute lymphoblastic leukemia cell lines with less in vitro neurotoxicity

**DOI:** 10.1186/s12935-020-01251-6

**Published:** 2020-05-15

**Authors:** Bryce Keenan, Rocio K. Finol-Urdaneta, Ashleigh Hope, John B. Bremner, Maria Kavallaris, Daniel Lucena-Agell, María Ángela Oliva, Jose Fernando Díaz, Kara L. Vine

**Affiliations:** 1 School of Chemistry and Molecular Bioscience, Molecular Horizons, Faculty of Science, Medicine and Health, University of Wollongong, Northfields Ave, Wollongong, NSW Australia; 2 Illawarra Health and Medical Research Institute, Northfields Ave, Wollongong, NSW 2522 Australia; 3 Electrophysiology Facility for Cell Phenotyping and Drug Discovery, Wollongong, NSW Australia; 4 Children’s Cancer Institute, Lowy Cancer Research Centre, UNSW, Sydney, NSW Australia; 5 ARC Centre of Excellence in Convergent Bio-Nano Science and Technology, UNSW, Sydney, NSW Australia; 6 School of Women’s and Children’s Health, Faculty of Medicine, UNSW Sydney, Sydney, NSW Australia; 7 Centro de Investigaciones Biológicas, Consejo Superior de Investigaciones Científicas, Madrid, Spain; 8 Centre for Oncology Education and Research Translation (CONCERT), Cancer Institute NSW Translational Cancer Research Centre, NSW, Sydney, Australia

**Keywords:** Chemotherapy, Multi-drug resistance, *N*-alkylisatin, P-glycoprotein, Neurotoxicity, Acute lymphoblastic leukemia

## Abstract

**Background:**

Drug resistance and chemotherapy-induced peripheral neuropathy continue to be significant problems in the successful treatment of acute lymphoblastic leukemia (ALL). 5,7-Dibromo-*N*-alkylisatins, a class of potent microtubule destabilizers, are a promising alternative to traditionally used antimitotics with previous demonstrated efficacy against solid tumours in vivo and ability to overcome P-glycoprotein (P-gp) mediated drug resistance in lymphoma and sarcoma cell lines in vitro. In this study, three di-brominated *N*-alkylisatins were assessed for their ability to retain potency in vincristine (VCR) and 2-methoxyestradiol (2ME2) resistant ALL cell lines. For the first time, in vitro neurotoxicity was also investigated in order to establish their suitability as candidate drugs for future use in ALL treatment.

**Methods:**

Vincristine resistant (CEM-VCR R) and 2-methoxyestradiol resistant (CEM/2ME2-28.8R) ALL cell lines were used to investigate the ability of *N*-alkylisatins to overcome chemoresistance. Interaction of *N*-alkylisatins with tubulin at the the colchicine-binding site was studied by competitive assay using the fluorescent colchicine analogue MTC. Human neuroblastoma SH-SY5Y cells differentiated into a morphological and functional dopaminergic-like neurotransmitter phenotype were used for neurotoxicity and neurofunctional assays. Two-way ANOVA followed by a Tukey’s post hoc test or a two-tailed paired *t* test was used to determine statistical significance.

**Results:**

CEM-VCR R and CEM/2ME2-28.8R cells displayed resistance indices of > 100 to VCR and 2-ME2, respectively. CEM-VCR R cells additionally displayed a multi-drug resistant phenotype with significant cross resistance to vinblastine, 2ME2, colchicine and paclitaxel consistent with P-gp overexpression. Despite differences in resistance mechanisms observed between the two cell lines, the *N*-alkylisatins displayed bioequivalent dose-dependent cytotoxicity to that of the parental control cell line. The *N*-alkylisatins proved to be significantly less neurotoxic towards differentiated SH-SY5Y cells than VCR and vinblastine, evidenced by increased neurite length and number of neurite branch points. Neuronal cells treated with 5,7-dibromo-*N*-(*p*-hydroxymethylbenzyl)isatin showed significantly higher voltage-gated sodium channel function than those treated with *Vinca* alkaloids, strongly supportive of continued action potential firing.

**Conclusions:**

The *N*-alkylisatins are able to retain cytotoxicity towards ALL cell lines with functionally distinct drug resistance mechanisms and show potential for reduced neurotoxicity. As such they pose as promising candidates for future implementation into anticancer regimes for ALL. Further in vivo studies are therefore warranted.

## Background

Acute lymphoblastic leukemia (ALL) is a cancer of the blood and bone marrow. It is the most common childhood cancer, and its incidence is expected to increase worldwide [1, 2]. Over the last 40 years, advances in the development of novel anti-microtubule agents, refinements in multi-agent chemotherapy regimens and precision medicine approaches have resulted in a dramatic improvement in survival rates of children with ALL [3]. However, the emergence of drug resistance remains a pertinent obstacle to successful treatment and accounts for ~ 20% of ALL treatment failures [4]. ALL is a polyclonal disease and mutations that confer a survival advantage in cellular subclones may be selected by chemotherapy, thereby promoting treatment resistance and subsequent patient relapse [5]. There has been minimal progress in treating relapsed disease for the last two decades highlighting the need for new approaches to improve the chances for cure for patients with relapsed and refractory ALL.

The current approach to treating relapsed ALL involves many of the same drugs used for newly diagnosed patients; however they are frequently delivered at increased doses or using alternative schedules. The *Vinca* alkaloid vincristine (VCR) is a standard component in every combination chemotherapy regimen used to treat ALL [5–7]. VCR is an antimitotic agent that targets the β-tubulin subunit of αβ-tubulin heterodimers, effectively destroying mitotic spindles and inhibiting cancer cell division through microtubule depolymerization. Though VCR is a potent antineoplastic agent, its clinical use is limited by a number of factors related to the development of resistance [8, 9] and off-target neurotoxicity [10–12]. Resistance to microtubule-targeted drugs, such as VCR, can be mediated by several mechanisms including the overexpression of transmembrane P-glycoprotein (P-gp), a member of the ATP-binding cassette (ABC) family [13, 14]. P-gp acts as a broad-spectrum drug efflux transporter which reduces the ability of cytotoxic agents to accumulate to therapeutic concentrations in the intracellular environment. Other ABC transporters such as multi-drug resistance-associated protein 1 (MRP1) and breast cancer resistance protein (BCRP) can also play a role [15]. Additionally, conformational changes in the drug target, such as mutations in the tubulin binding site, alterations in tubulin isotypes and/or altered microtubule polymer levels are key contributors to VCR drug resistance [16, 17]. Current evidence suggests that these cellular changes favour more stable microtubules or affect the microtubule dynamics such that VCR effectiveness is decreased [18]. Altogether, this calls for the development of novel drugs that can circumvent drug-efflux mechanisms and/or changes in the tubulin target as an alternative therapeutic approach.

Molecules targeting the colchicine binding site on tubulin may provide an opportunity to overcome such ABC transporter-mediated drug resistance [19, 20]. In fact, mounting evidence indicates that compounds interacting with the colchicine binding site are also less sensitive to other clinically-relevant mechanisms of resistance, including overexpression of the βIII-tubulin isotype [21]. Colchicine binds at the interface between the α- and β-tubulin monomers and prevents α-tubulin from stacking onto the β-tubulin β-sheet, thereby inhibiting microtubule assembly [22]. While colchicine itself is not used as an anticancer agent due to off-target toxicities, a large number of structurally diverse tubulin inhibitors targeting the colchicine site are currently being evaluated in preclinical and clinical trials for cancer with promising outcomes [23]. Interestingly, compounds bearing an indole ring form part of a growing number of antimitotic compounds that bind to the colchicine site on tubulin [24]. A related class of compounds that show great potential in mitigating multi-drug resistance (MDR), possibly through binding to the colchicine site or other sites [25] are those based on the isatin (1*H*-indole-2,3-dione) scaffold [26, 27]. Synthesis and subsequent cytotoxicity evaluation of a library of 5,7-dibominated *N*-alkylated isatins by our group found them to elicit potent cytotoxic activity against a range of human cancer cell lines in the sub-micromolar range [28, 29]. Tubulin binding experiments demonstrated that they destabilize microtubule growth, similar to the *Vinca* alkaloids and colchicine, by inhibiting microtubule polymerization [28, 29]. Interestingly, these *N*-alkylisatins retained potency against P-gp-overexpressing cell lines including both doxorubicin resistant uterine sarcoma and vinblastine resistant lymphoma cell lines [30]. While preliminary investigations into the potential binding site of the *N*-alkylisatins on tubulin have been performed, it remains unknown whether this class of molecules bind at a known site, or exploit a novel site on tubulin.

**Fig. 1:**
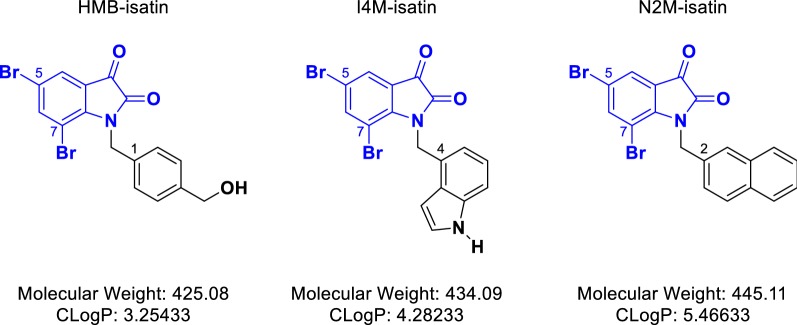
Chemical Structures of the three *N*-alkylisatin derivatives. Derivation of the 5,7-dibromoisatin scaffold (blue) led to the development of potent antimitotic *N*-alkylated analogues: 5,7-dibromo-*N*-(*p*-hydroxymethylbenzyl)isatin (HMB-isatin), 5,7-dibromo-*N*-(1*H*-indol-4-ylmethyl)isatin (I4M-isatin) and 5,7-dibromo-*N*-(naphthalene-2-ylmethyl)isatin (N2M-isatin)

In order to investigate the ability of *N*-alkylisatins (Fig. 1) to overcome chemoresistance in ALL and to further elucidate molecular interactions with tubulin, we conducted cytotoxicity assays using VCR and 2-methoxyestradiol (2ME2) resistant ALL cell lines, the latter harbouring single-base mutations in the colchicine and paclitaxel binding sites of class I β-tubulin. We further studied the interaction of *N*-alkylisatins with tubulin at the the colchicine-binding site using competition assays. Finally, given that vincristine-induced peripheral neuropathy continues to be a dose-limiting factor in ALL chemotherapy, the neurotoxicity of three *N*-alkylisatins was studied in vitro using neurite outgrowth assays and neurofunctional electrophysiological experiments in order to further assess their suitability as candidate drugs for future use in ALL treatment.

## Materials and methods

### Cell lines and culture conditions

The CCRF-CEM human T-cell acute lymphoblastic leukemia (T-ALL) cell line (CEM) [31] was originally supplied by P. Slowiaczek (Ludwig Institute, Sydney, Australia). The vincristine-resistant (CEM-VCR R) and 2-methoxyestradiol-resistant (CEM/2ME2-28.8R) sublines were prepared by continuous exposure to either VCR or 2ME2 respectively, as described in [13, 17]. ALL cell lines were routinely cultured in RPMI-1640 media containing 2 mM l-glutamine (Sigma-Aldrich, Sydney, Australia), supplemented with 10% (v/v) heat-inactivated fetal calf serum (FCS) (Sigma-Aldrich). The human neuroblastoma SH-SY5Y cell line (American Type Culture Collection, CRL-2266) was maintained in DMEM/F12 media (Sigma-Aldrich) supplemented with 10% (v/v) heat-inactivated FCS. All cell lines were confirmed negative for mycoplasma contamination and cultured at 37 °C in a 95% humidified air atmosphere, containing 5% CO_2_. Culturing of cells for experimental use did not exceed 20 passages.

### Cytotoxins

The *N*-alkylisatins; 5,7-dibromo-*N*-(*p*-hydroxymethylbenzyl)isatin (HMB-isatin), 5,7-dibromo-*N*-(1*H*-indol-4-ylmethyl)isatin (I4M-isatin), 5,7-dibromo-*N*-(naphthalene-1-ylmethyl)isatin (N1M-isatin) and 5,7-dibromo-*N*-(naphthalene-2-ylmethyl)isatin (N2M-isatin) were synthesized by TCG Lifesciences Pty Ltd (Kolkata, India) following methodology described by our group previously [28, 29]. Colchicine, paclitaxel and vinblastine sulfate were purchased from Sigma-Aldrich (St. Louis, MO, USA). Vincristine sulfate and 2-methoxyestradiol were purchased from Abcam (Sydney, Australia) and Selleck Chem (Sydney, Australia), respectively. All compounds were solubilized in 100% dimethyl sulfoxide (DMSO) (Sigma-Aldrich, St. Louis, MO, USA) and diluted in the appropriate media/buffer for downstream assays as described below.

### Cell viability (MTS) assay

Cytotoxicity of drugs was determined using the CellTiter 96 Aqueous One Solution Cell Proliferation (MTS) Assay (Promega, USA) in flat-bottomed 96-well microplates. Cells were centrifuged at 300×*g* for 5 min at room temperature, resuspended in fresh growth media and seeded at 10,000 cells/well for CEM and CEM-VCR R cells, and 20,000 cells/well for CEM/2ME2-28.8R cells (90 μL) based on differences in cell growth properties (Additional file 1: Figure S1). Cells were then incubated for 24 h under standard culture conditions before the addition of cytotoxins. DMSO stocks of cytotoxins were serially diluted in media to give 10 × working stock (10% DMSO). This was further diluted to give a final drug concentration of cytotoxin in 1% DMSO. Following treatment, cells were further incubated at 37 °C for 48 h. At this time-point, 20 μL of MTS reagent was added to each well and incubated for 3 h at 37 °C, allowing for sufficient color change. Optical density (OD) was subsequently measured at 490 nm using the Spectrostar Nano Microplate Reader (BMG Labtech, Mornington, Australia). Cytotoxicity of drugs was calculated from dose response curves generated in GraphPad Prism v7.0 (Graphpad Software Inc., USA) and reported as cell viability (percent of DMSO control). IC_50_ values were calculated from sigmoidal dose response curves and defined as the concentration required to inhibit the metabolic activity of 50% of the cell population. The resistance index (RI) was calculated to evaluate the degree of acquired resistance of each cell line to the various drug treatments and was determined using the following equation: $RI = \frac{{IC50 \,\left( {resistant\,subline} \right)}}{{IC50 \,\left( {sensitive\,subline} \right)}}$. Cellular morphological changes upon drug exposure were also tracked using the IncuCyte ZOOM (Essen Bioscience, Ann Arbor, MI, USA) and real-time quantitative live-cell analysis.

### Rhodamine 123 efflux assay

The rhodamine 123 (R123) efflux assay was carried out as described in [30] with slight alterations to the protocol. Briefly, P-gp was probed on CEM-VCR R cells using the fluorescent P-gp substrate, R123, in the absence or presence of cyclosporine A (CSA), a specific P-gp inhibitor. Cells were seeded (10,000 cells/well) in 96-well microplates at a volume of 100 μL and incubated at 37 °C for 24 h. The microplate was then centrifuged at 300×*g* and supernatant was removed. Cells were then resuspended in media containing R123 (5 μM) in the absence or presence of CSA (20 μM) or DMSO vehicle control (final concentration 1% v/v). Cells were incubated at 37 °C for a further 20 min in the dark. Culture media was removed via centrifugation and the cells washed with ice-cold PBS. Cells were successively spun at 300×*g*, supernatant removed and the cell pellet lysed with 1% Triton-X (100 µL). An aliquot (80 μL) of the cell lysate was then added to wells of a 96-well microplate. Intracellular fluorescence was subsequently measured using a FLUOstar Optima Microplate Reader (BMG Labtech, Ortenburg, Germany) using an excitation wavelength of 485 nm, and emission wavelength of 530 nm.

### Tubulin binding assays

The tubulin target site of the *N*-alkylisatins and their associated binding affinity constant (K_b_) were determined by competitive assay using a fluorescent colchicine analogue 2-methoxy-5-(2,3,4-trimethoxyphenyl)-2,4,6-cycloheptatrien-1-one (MTC) that reversibly binds to the colchicine-binding site as described in [32]. Equal amounts of pure tubulin and MTC (10 µM) were mixed in a phosphate-based buffer (10 mM phosphate buffer pH 7, 1 mM EDTA, 1.5 mM MgCl_2_, 0.1 mM GTP), and incubated for 30 min at 25 °C with increasing concentrations up to 50 μM of the respective drug prior to the addition of 10 μM MTC. Afterwards, displacement of MTC bound to tubulin was monitored by changes in fluorescence intensity using a Varioskan^®^ Flash plate reader (ThermoFisher Scientific) (excitation and emission wavelengths of 370 nm and 423 nm, respectively; 100 ms of exposure time with a bandwidth of 12 nm). Tubulin and MTC alone served as negative controls for fluorescence normalization. Fluorescence intensity data were converted to fractional saturation and binding constants were obtained using Equigra V5.0 as described in [33].

### Neurite outgrowth assay

SH-SY5Y cells were seeded at a density of 5000 cells/well (100 µL) in 96-well plates and incubated for 24 h (37 °C, 95% humidity, 5% CO_2_). Media (DMEM/F12 + 10% FCS) was replaced with DMEM/F12 + 1% FCS + 10 μM retinoic acid (RA) (Sigma-Aldrich, Sydney, Australia) to induce differentiation into neuronal-like phenotype [34]. RA supplemented media (DMEM/F12 + 1% + FCS + 10 µM RA) was replaced every 48 h. Following sufficient neurite growth (approximately 96 h after initial addition of RA), various concentrations (1 nM, 10 nM and 100 nM) of vincristine, vinblastine, HMB-isatin, I4M-isatin or N2M-isatin were added to the cells and incubated for a further 48 h. Neurite outgrowth analysis was performed using the NeuroTrack analysis software on the IncuCyte ZOOM (Essen Bioscience). The algorithm masks and quantifies each image and returns neuronal cell metrics including neurite length and number of branch points per cell body [35].

### Automated patch-clamp electrophysiology

SH-SY5Y cells were detached from 6 well dishes using TrypLE (ThermoFisher Scientific) and re-suspended in cold external recording solution as described in [36]. Cells were kept in suspension by automatic pipetting at 4 °C. Patch-clamp measurements were performed on an NPC-16 Patchliner (Nanion Technologies, Munich, Germany) using single and multi-hole medium resistance NPC-16 chips with an average resistance of 3.1 and 1.1 MΩ, respectively. Recordings were made in the perforated-patch configuration with internal solution consisting of (in mM): 60 CsF, 50 CsCl, 20 NaCl, 10 HEPES, 10 EGTA (285 ± 3 mOsm; pH7.2 with CsOH); or 60 KF, 50 KCl, 20 NaCl, 10 HEPES, 10 EGTA (285 ± 3 mOsm; pH7.2 with KOH) supplemented with the membrane perforating agent Escin (25 µg/mL; Sigma-Aldrich). The extracellular solution contained (in mM): 140 NaCl, 5 Glucose, 4 KCl, 2 CaCl_2_, 1 MgCl_2_, 10 HEPES (298 ± 3 mOsm; pH7.4 with NaOH). Seal formation was enhanced by brief treatment with SE solution (in mM): NaCl 80, KCl 3, MgCl_2_ 10, CaCl_2_ 35, HEPES 10 (298 ± 3 mOsm, pH = 7.3 adjusted with NaOH) until stable seals were obtained and replaced with standard external solution. Solutions were filtered through 0.2 μm membranes. Recording seal resistance was > 500 MΩ and access resistance was < 8 MΩ in the perforated patch configuration.

Chip and whole-cell capacitance were fully compensated, and series resistance compensation was applied via Auto Rs Comp function. Recordings were acquired at 50 kHz with the low-pass filter set to 10 kHz with Patchmaster (HEKA Elektronik, Lambrecht/Pfalz, Germany) and stored on a computer running PatchControlHT software (Nanion Technologies GmbH, Munich, Germany). Leak subtraction was performed automatically using a P/4 procedure following the test pulse. Offline analysis was performed using Igor Pro-6.37 (WaveMetrics Inc.), GraphPad Prism 7 (Molecular Devices) and Microsoft Excel. The pulse protocol used to evaluate voltage dependent sodium conductance consisted of a family of 25 ms test pulses from −60 to 60 mV (+10 mV increments) following a 100 ms pre-pulse to −120 mV. The holding potential (Vh) was −80 mV and stimulation was performed at 0.1 Hz. All recordings were conducted at room temperature. A 300 nM solution of tetrodotoxin, (TTX, Sigma Aldrich) was applied after IV recordings to determine the relative contribution of TTX-sensitive Na_v_ channels.

To examine the voltage-dependent parameters of the sodium current, peak current amplitude from 25 ms test pulse potentials (100 ms pre-pulse to −120 mV, followed by step depolarization from −60 to 60 mV, ∆10 mV, Vh: −80 mV) were measured. Peak IV curves were fitted using: I_(V)_ = (V − V_rev_)*G_max_/[1 + exp^(V0.5−Vslope)^], where I is the macroscopic current, V is the command potential, V_rev_ is the reversal potential (mV), G_max_ is the maximal conductance (S), V_0.5_ is the half-activation potential (mV), and Vslope is the slope factor (mV/e-fold).

### Statistical analysis

Statistical analysis of IC_50_ values and neurite outgrowth assays were calculated using a two-way ANOVA followed by a Tukey’s post hoc test. A two-tailed, paired t-test was applied to compare intracellular levels of R123 between resistant and parental cells, as well as CSA-treated and non-treated cells. A two-tailed, paired t-test was also applied to compare current density between retinoic acid differentiated cells to all other experimental groups. All calculations were performed using GraphPad Prism v7.0 (Graphpad Software Inc., USA). Statistically significant results were represented by p < 0.05 (α-value of 0.05).

## Results

### Cell viability and efflux assays

We have previously demonstrated that 5,7-dibromo-*N*-alkylisatins with varied substituents at the isatin nitrogen retain potency in drug resistant human lymphoma (U937) and uterine sarcoma (MES-SA) MDR cancer cell lines in vitro [30]. In order to determine the ability of three 5,7-dibromo-*N*-alkylisatins (Fig. 1) to retain cytotoxicity against ALL cell lines with differing mechanisms of resistance, the cytotoxic effects of commercial antimitotic drugs: vincristine (VCR), vinblastine (VBL), 2-methoxyestradiol (2ME2), colchicine and paclitaxel and the *N*-alkylisatins: HMB-isatin, I4M-isatin and N2M-isatin were tested against the VCR-resistant (CEM-VCR R) and 2ME2-resistant (CEM/2ME2-28.8R) ALL cell lines, and compared to the chemosensitive parental ALL cell line (CEM) using the MTS cell proliferation assay.

**Fig. 2:**
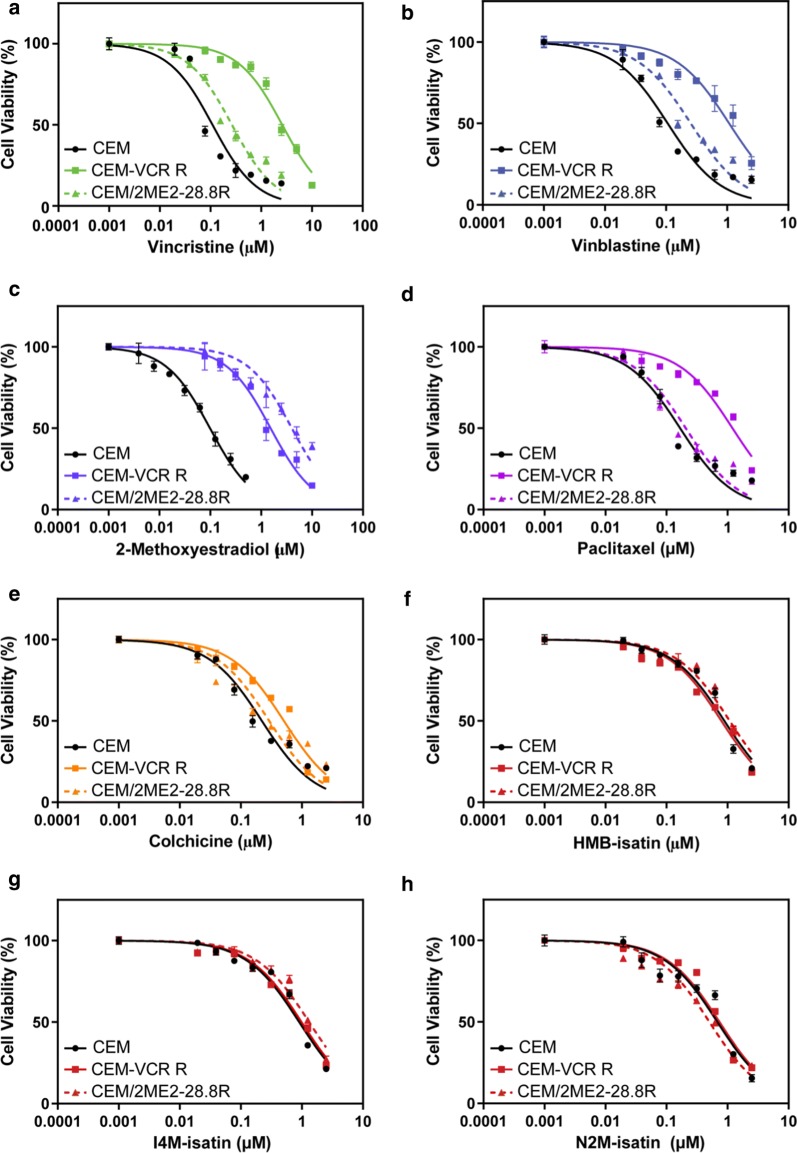
Dose-response curves for parental (CEM), vincristine-resistant (CEM-VCR R) and 2ME2-resistant (CEM/2ME2-28.8R) ALL cell lines treated with varying concentrations of commercial drugs (**a–e**) or *N*-alkylisatins (**f–h**). CEM (black), CEM-VCR R (solid) and CEM-2ME2/28.8R (dashed) cells were incubated with increasing concentrations of drug at 37 °C for 48 h. Cells were then assessed for metabolic activity by addition of MTS reagent and the OD measured at 490 nm. Dose–response curves were generated using GraphPad Prism v7.0. Values are the mean of triplicates (± SD)

CEM-VCR R cells displayed a 101.4-fold (t_6_= 4.869, p < 0.005) decrease in sensitivity to VCR and were significantly cross-resistant to VBL, 2ME2, colchicine and paclitaxel (Table 1). Treatment of CEM/2ME2-28.8R cells with 2ME2 revealed a 106.5-fold (t_8_ = 4.529 p < 0.05) decrease in drug sensitivity. Interestingly, these cells did not demonstrate cross-resistance to VCR, VBL, colchicine or paclitaxel. Notably, both CEM-VCR R and CEM/2ME2-28.8R resistant cell lines treated with HMB-isatin, I4M-isatin or N2M-isatin showed a negligible change in IC_50_ values (1.0–1.4-fold increase, p > 0.05) after 48 h compared to their respective parental cells (Fig. 2). A Two-Way Analysis of Variance (2-way ANOVA) found no significant difference in the viability of CEM, CEM-VCR R or CEM/2ME2-28.8R cells treated with increasing concentrations of the *N*-alkylisatins, irrespective of differences in their molecular weight, lipophilicity or N1 substitution patterns.

**Table 1:** IC_50_ (µM) values and fold decrease in drug sensitivity (RI) of parental (CEM) versus resistant (CEM-VCR R and CEM/2ME2 28.8R) human acute lymphoblastic leukemia cell lines treated with microtubule targeting agents

IC_50_ (µM) ± SD^a^				Resistance Index (RI)^b^	
Compound	CEM	CEM-VCR R	CEM/2ME2-28.8R	CEM-VCR R:CEM	CEM/2ME2-28.8 R:CEM
Vincristine	0.08 (± 0.05)	8.11 (± 1.62)	0.41 (± 0.19)	101.4 < 0.05	5.1 > 0.05
Vinblastine	0.13 (± 0.06)	1.12 (± 0.16)	0.39 (± 0.13)	8.6 < 0.05	3.0 > 0.05
2-Methoxy-estradiol	0.08 (± 0.04)	4.07 (± 1.34)	8.52 (± 1.38)	50.9 < 0.05	106.5 < 0.05
Colchicine	0.32 (± 0.21)	1.01(± 0.36)	0.56 (± 0.19)	3.2 < 0.05	1.8 > 0.05
Paclitaxel	0.15 (± 0.09)	1.11 (± 0.36)	0.25 (± 0.12)	7.4 < 0.05	1.7 > 0.05
HMB-isatin	1.02 (± 0.32)	1.07 (± 0.37)	1.29 (± 0.41)	1.0 > 0.05	1.3 > 0.05
I4M-isatin	1.07 (± 0.44)	1.26 (± 0.19)	1.16 (± 0.46)	1.2 > 0.05	1.1> 0.05
N2M-isatin	0.36 (± 0.19)	0.49 (± 0.36)	0.46 (± 0.32)	1.4 > 0.05	1.3 > 0.05

^a^Values are the mean of three independent experiments, performed in triplicate (n = 9) ± SD

^b^The RI was calculated to determine the degree of acquired resistance of each cell line to the various drug treatments and was determined using the following equation:$RI = \frac{{IC50 \left( {resistant\,subline} \right)}}{{IC50 \left( {sensitive\,subline} \right)}}$. P-values < 0.05 were considered significant

**Fig. 3:**
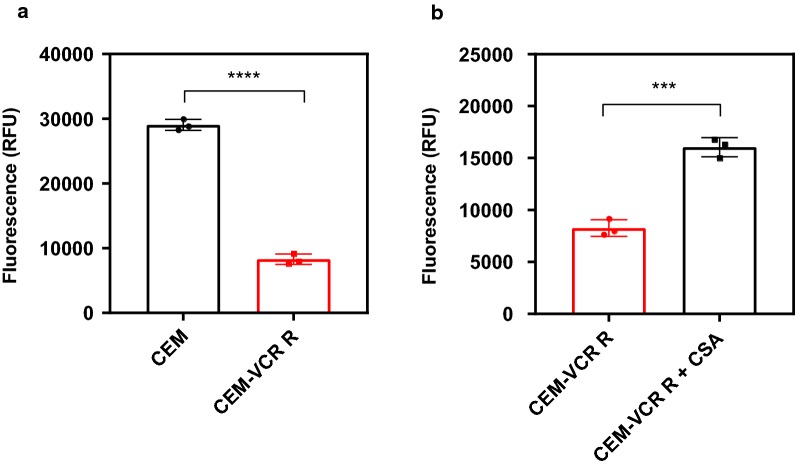
Levels of intracellular rhodamine 123 (R123) in CEM and CEM-VCR R cell lines in the presence and absence of cyclosporine A (CSA). **a** Parental (CEM) and VCR-resistant cells (CEM-VCR R) were incubated with R123 for 20 min before being washed with PBS and lysed with 1% Triton-X. **b** CEM-VCR cells were incubated with R123 in the presence or absence of 20 µM Cyclosporin A (CSA), a specific P-gp inhibitor, for 20 min before undergoing subsequent washing and lysis. Intracellular fluorescence was subsequently measured using a FLUOstar Optima Microplate Reader using an excitation wavelength of 485 nm, and emission wavelength of 530 nm. Mean ± SD, *n *= 3. ***P < 0.001, ****P < 0.0001

Overexpression of the P-gp efflux transporter has previously been reported as one of the primary mechanisms of resistance in the CEM-VCR R cell line [13, 37, 38]. In order to confirm that P-gp was functional, we monitored the efflux of a fluorescent Rhodamine 123 (R123), a known P-gp substrate. Intracellular fluorescence was significantly increased by 3.5-fold in CEM cells compared to CEM-VCR R cells (t_4_ = 30.97, p < 0.05) (Fig. 3a). To further confirm that R123 extrusion from CEM-VCR R cells was the result of P-gp mediated efflux, cells were incubated with R123 in the absence or presence of cyclosporin A (CSA), a specific P-gp inhibitor. Here, CEM-VCR R cells incubated with R123 showed a twofold increase in intracellular fluorescence in the presence of CSA (t_4_ = 11.08, p < 0.05) (Fig. 3b).

### Competition binding assays

**Fig. 4:**
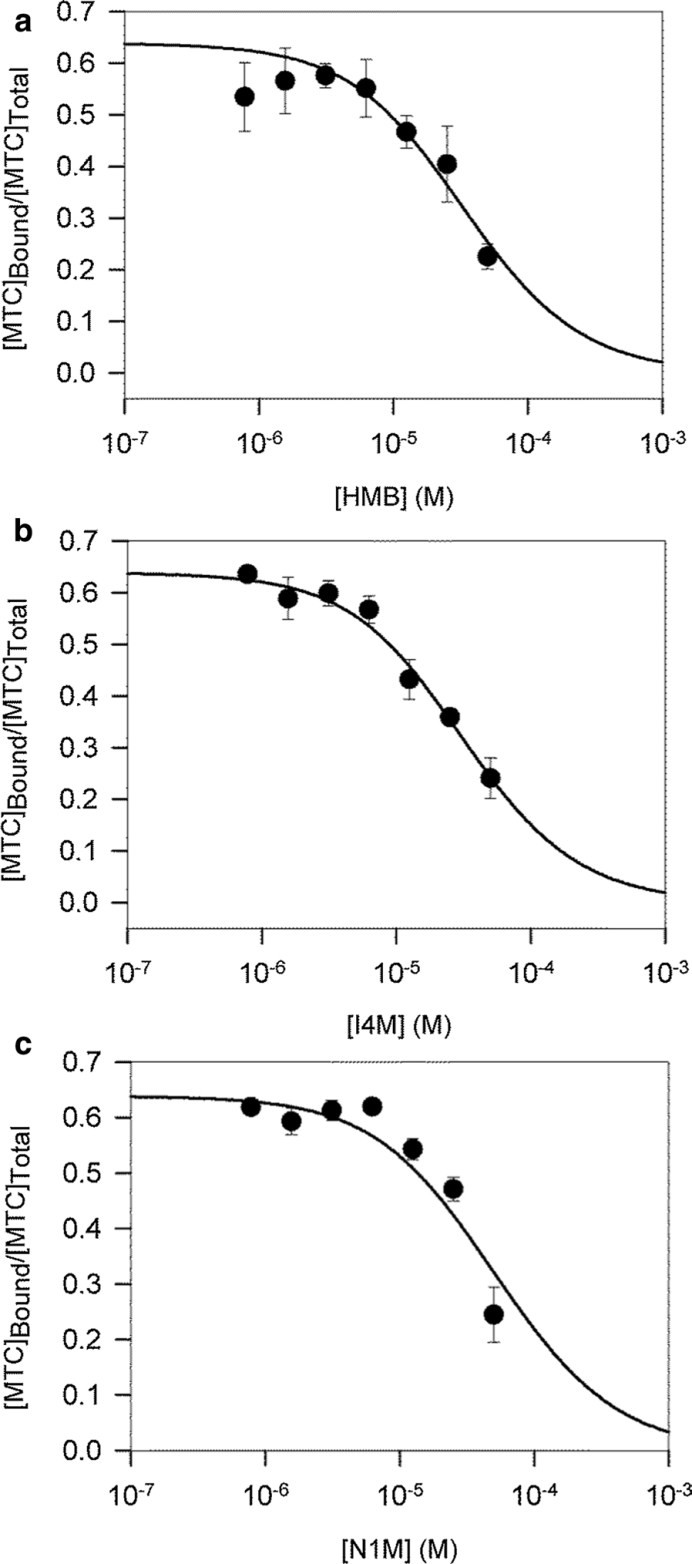
Displacement of MTC from the colchicine-binding site by **a** HMB-isatin, **b** I4M-isatin and **c** N1M-isatin. MTC fractional saturation values were calculated based on the concentration of MTC binding sites (0.8 per tubulin dimer) and the MTC binding constant (4.7 × 10^5^ M^−1^). Solid line is the best fit to a single site competition model as calculated by EQUIGRA5. Values are the mean of triplicates ± SEM

Pioneering work by our group has revealed that 5,7-dibromo-*N*-alkylisatins inhibit tubulin polymerization, resulting in the accumulation of cells with atypical cell morphologies, fragmented microtubules and abnormal mitotic spindles [28, 29]. To obtain further insight into the molecular interaction of our *N*-alkylisatins with tubulin, and because the colchicine site is a major binding pocket for many indole, quinolone and thiophene-based microtubule destabilizers [23], we investigated the ability of HMB-isatin, I4M-isatin and N1M-isatin (where the naphthalene is instead attached via the C1 position instead of the C2 position) to bind to this site using a reversible fluorescent colchicine analogue MTC [32]. All compounds were shown to compete with similar affinities (within the experimental error) to the colchicine analog, displacing it from its binding site and reducing its fractional saturation, consistent with binding to the colchicine site (Fig. 4 and Table 2).

**Table 2:** Binding affinity values for HMB-isatin, I4M-isatin and N1M-isatin compared with reference compounds of the colchicine site

Compound	Binding affinity (M^−1^) ± SEM^a^
Colchicine	1.16 × 10^7^ (37 °C) [70]
Podophyllotoxin	1.8 × 10^6^ [71]
MTC	4.7 × 10^5^ [72]
HMB-isatin	2.7 ± 1.1 × 10^5^
I4M-isatin	3.4 ± 1.5 × 10^5^
N1M-isatin	1.4 ± 0.2 × 10^5^(× 10^4^)

^a^Values (K_b_) are the mean of three independent experiments performed in triplicate (n = 9) ± SEM

### Neurite outgrowth assay

**Fig. 5:**
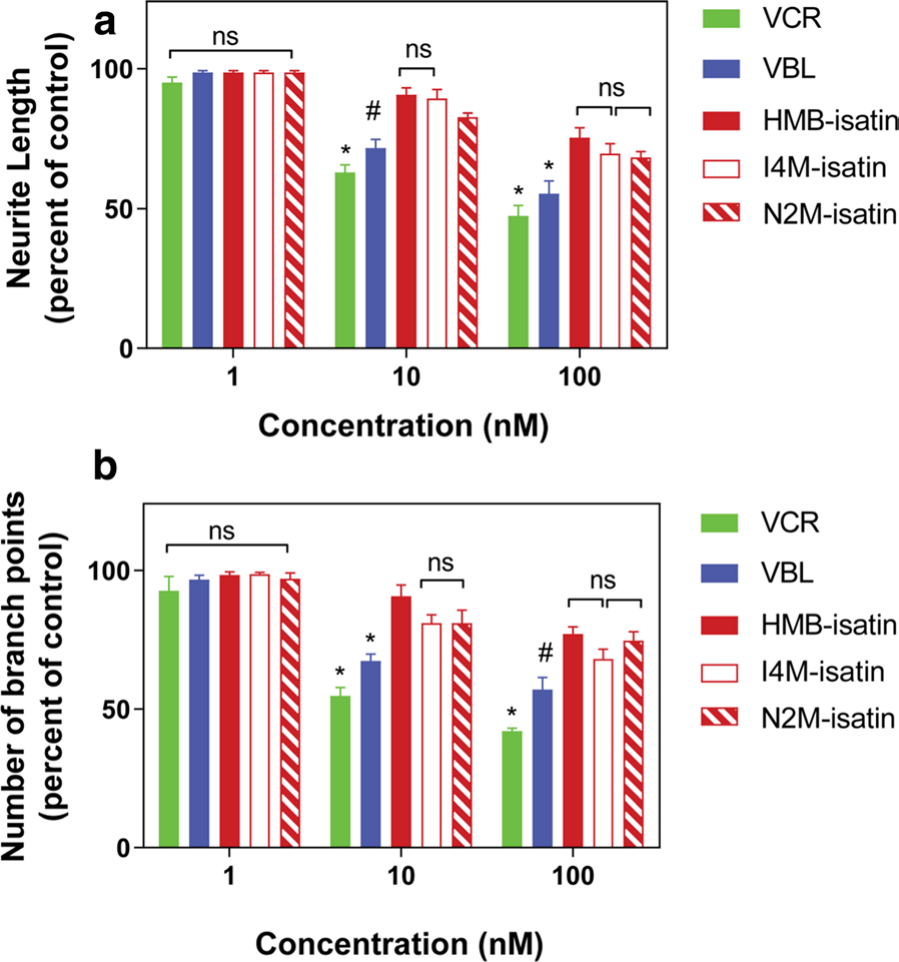
Effect of *N*-alkylisatins and Vinca alkaloids on SH-SY5Y neurite outgrowth and number of neurite branch points. SH-SY5Y neuroblastoma cells were incubated with 10 μM retinoic acid for 96 h to induce differentiation of cells into a mature dopaminergic-like neurotransmitter phenotype. Cells were then treated with increasing concentrations (1–100 nM) of *N*-alkylisatin (HMB-isatin, I4M-isatin or N2M-isatin) or *Vinca* alkaloid (vincristine; VCR or vinblastine; VBL) for 48 h. Changes in **a** neurite length or **b** number of neurite branch points were analyzed using the IncuCyte ZOOM, and measured using NeuroTrack analysis software. Mean ± SD, *n *= 3. NS = not significant, *P < 0.001,^#^P < 0.01

**Fig. 6:**
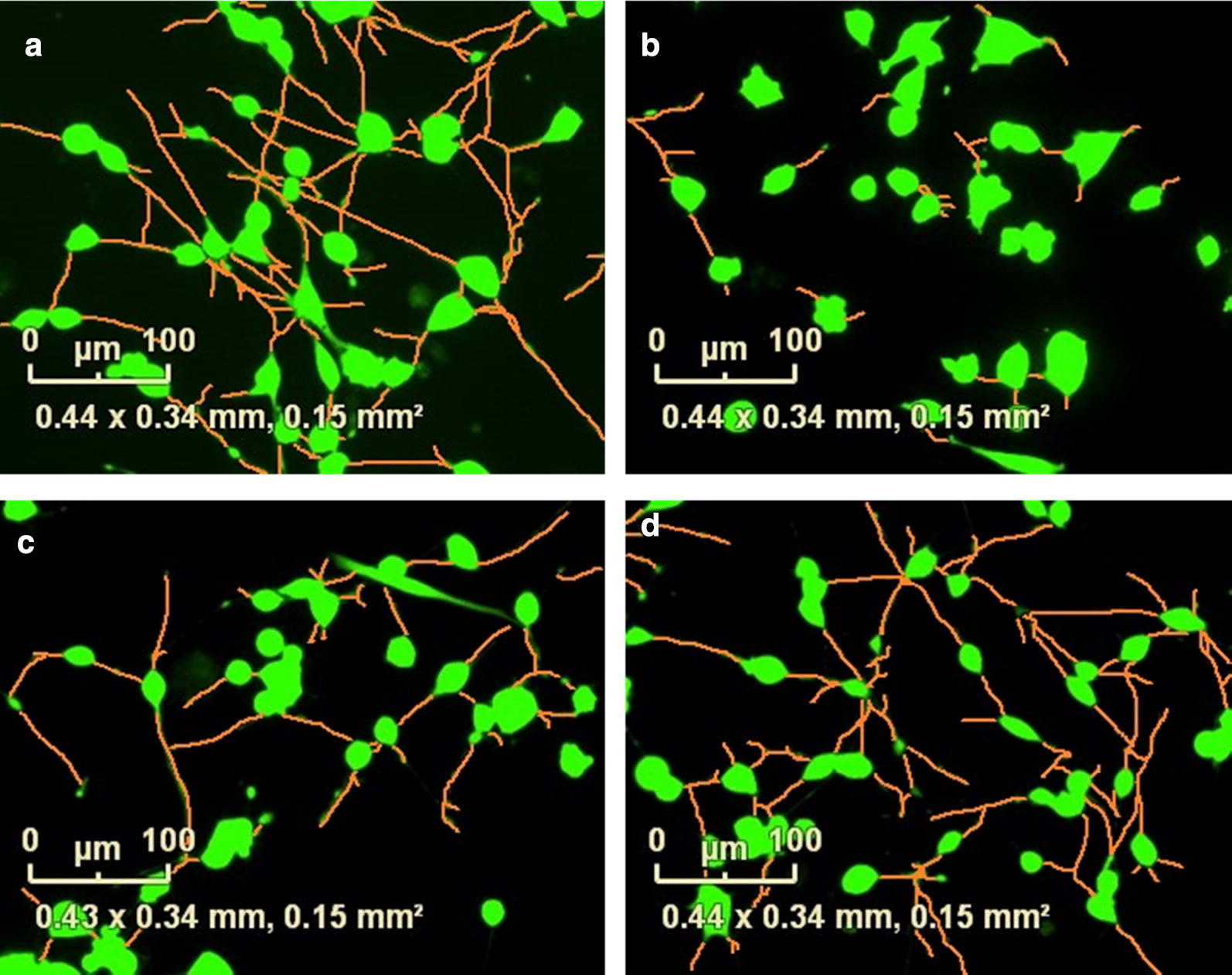
*Vinca* alkaloids significantly alter the neurite morphology of retinoic acid differentiated SH-SY5Y cells. Automated segmentation of neurites (orange) and calcein AM stained cell body clusters (green) of retinoic acid-differentiated SH-SY5Y cells after overnight incubation with **a** 1% DMSO vehicle control, or 10 nM **b** vincristine, **c** vinblastine or **d** HMB-isatin. Images we obtained using Neurotrack and the IncuCyte^®^ live-cell analysis system

Peripheral neuropathy is the principal toxicity associated with the use of the *Vinca* alkaloids, especially in younger children with ALL [39]. Immortalized cell lines derived from CNS tissue are powerful tools for elucidating the mechanism of action of neurotoxic chemicals [40] as they recapitulate key aspects of neuronal function and overcome some of the limitations associated with the use of ex vivo and in vivo models such as heterogeneity (genetic variability) which obscures interpretation of mechanistic data. To compare the potential neurotoxic effect of our *N*-alkylisatins to VCR and VBL we performed neurite outgrowth assays using retinoic acid (RA) differentiated SH-SY5Y cells and measured alterations in neurite length and number of branch points (Figs. 5, 6 and Additional file 1: Figure S3). RA differentiated SH-SY5Y cells were treated with sub-IC_50_ concentrations of the *Vinca* alkaloids or *N*-alkylisatins and compared to vehicle control treated cells. No significant differences in neurite length were observed between any of the drugs and the control group at 1 nM (p > 0.05) (Fig. 5a). Cells treated with VCR and VBL at 10 nM and 100 nM however, showed a significant (p < 0.05) reduction in neurite length (56% and 45% for 100 nM VCR and VBL, respectively) compared to those vehicle treated. Conversely, treatment with HMB-isatin, I4M-isatin and N2M-isatin at 10 nM demonstrated 7% –15% reduction in neurite length and thus appeared significantly less neurotoxic than VCR and VBL (p < 0.05) at the same concentration. Similarly, SH-SY5Y cells treated with 100 nM HMB-isatin, I4M-isatin or N2M-isatin displayed 25%–35% reduction in neurite length, compared to the no drug control, but significantly less than VCR and VBL (P < 0.05).

To further interrogate neurotoxicity, numbers of neurite branch points were examined following drug treatments (Fig. 5b). At 1 nM, all drugs exhibited minimal effect on the number of neurite branch points and were not shown to be significantly different compared to no drug controls (p > 0.05). VCR and VBL treatment at 10 nM caused a significant (p < 0.05) reduction in number of neurite branch points (46% and 33%, respectively) compared to vehicle treated control. At the same concentration HMB-isatin, I4M-isatin and N2M-isatin reduced the number of neurite branch points by only 8%–17% and were significantly different to VCR and VBL (p < 0.05). Treatment with VCR and VBL at 100 nM revealed a significant (p < 0.05) reduction in neurite branch points (55% and 45%, respectively) compared to no drug treated control. At the same concentration, HMB-isatin, I4M-isatin and N2M-isatin reduced branch points by only 25%–32% and, significantly less than VCR and VBL (p < 0.05). Upon completion of the assay, cell viability was immediately assessed by MTS assay. No significant differences in cell viability were identified across all drugs and concentrations tested (Additional file 1: Figure S2). Overall, RA differentiated SH-SY5Y cells were less affected after treatment with the *N*-alkylisatins compared to VCR and VBL at equivalent concentrations (p < 0.05), indicating a potential for lowered neurotoxicity.

### Functional assessment by automated patch-clamp electrophysiology

**Fig. 7:**
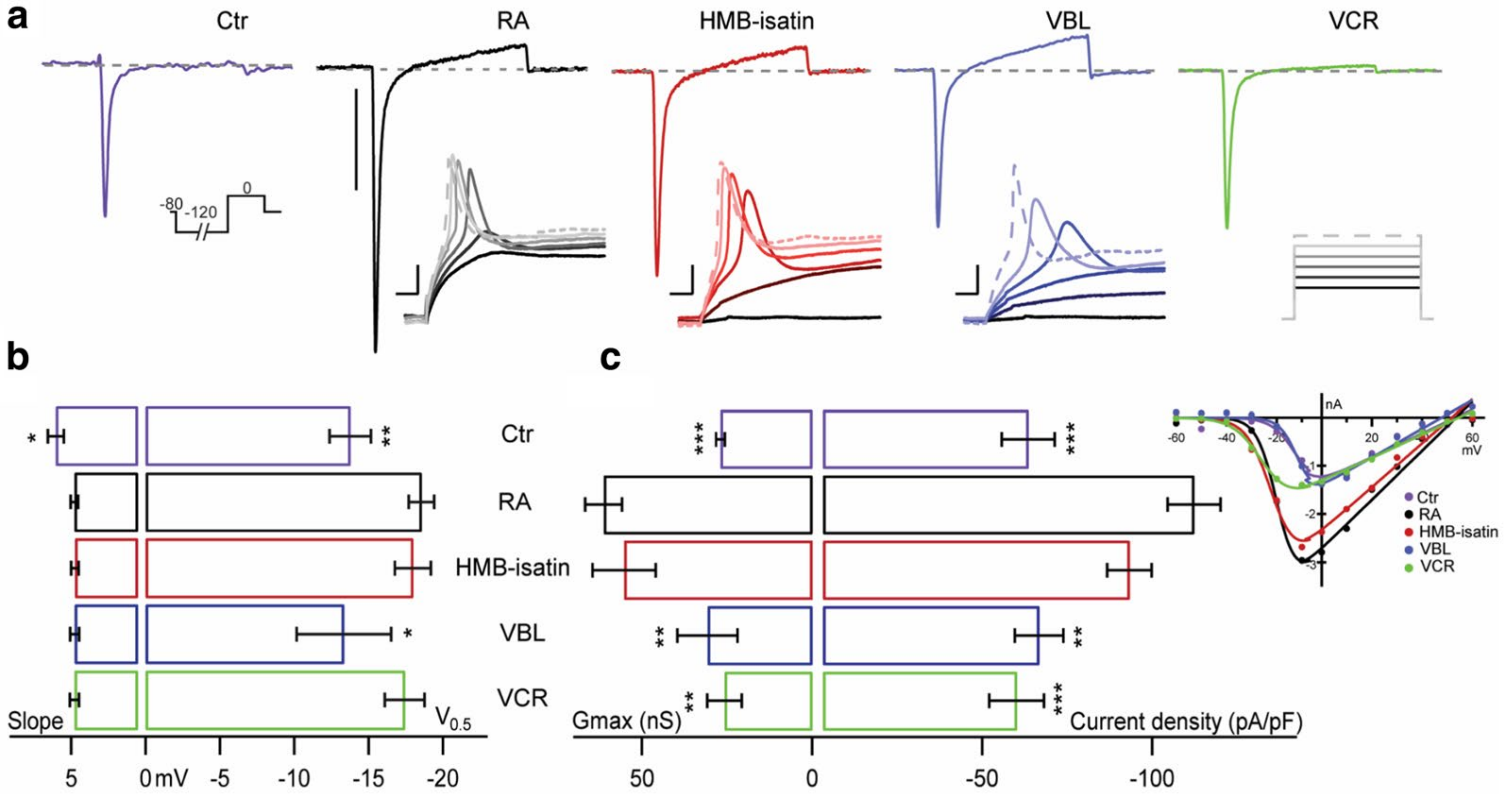
Cytotoxic drugs differentially affect the electrophysiological properties of SH-SY5Y RA differentiated cells. **a** Top traces depict whole cell voltage clamp recordings of endogenous currents from representative naïve SH-SY5Y cells (Ctr, purple), retinoic acid differentiated (RA, black), and RA-differentiated cells treated with 5 nM HMB-isatin (red), 5 nM vinblastine (VBL, blue) and 5 nM vincristine (VCR, green). Currents were elicited by 25 ms step depolarizations to 0 mV from a Vh: -80 mV preceded by a 100 ms –120 mV pre-pulse (inset under Ctr). Scale bar: 1 nA. The insets below RA, HMB-isatin and VBL show voltage traces in response to current injection (50–100 pA, ∆10 pA, inset under VCR) that elicited action potentials from their respective Vh. Scale bars are: 10 mV and 100 ms. **b** and **c** Voltage dependent parameters of inward sodium currents in all groups obtained from IV stimulation protocols from −60 to 60 mV (∆10 mV, Vh: −80 mV). IVs from representative cells are shown in the inset on the right. **b** Slope and activation half voltage. **c** Right: maximal conductance (Gmax) and inward current density estimated from the ratio of peak current amplitude observed at 0 mV to the cell membrane capacitance (pA/pF). Ctr: non differentiated; RA: Retinoic acid differentiated; HMB-isatin: 5,7-dibromo-*N*-(*p*-hydroxymethylbenzyl)isatin treated RA cells; VBL: vinblastine treated RA cells; VCR: vincristine treated RA cells (drugs were applied overnight at 10 nM). Mean ± SEM from n = 5–24 cells per condition. Unpaired t-test compares RA against all other groups with a 95% confidence interval. Data are presented as means ± SEMs (n represents the number of individual recordings). A two-tailed, paired t-test was applied to compare current density between retinoic acid differentiated cells to the other experimental groups. Data presented is compiled from 3 independent experiments involving cell differentiation and drug treatment

Neuronal morphology with neuritic-like processes in RA differentiated SH-SY5Y cells is associated with functional features that include transmembrane currents underlying a neuronal excitability phenotype [41–43]. Such cells lines can therefore serve as a tool in neurofunctional assays. Here, we compared the effects of overnight incubation with HMB-isatin, VCR and VBL on the electrophysiological properties of non-treated RA differentiated SH-SY5Y cells and naïve SH-SY5Y (non-RA differentiated) cells. Cells from all treatment groups were studied by automated patch clamp in the perforated patch configuration using standard solutions. In all cell groups we observed rapidly activating and inactivating inward currents followed by modest delayed outward currents in response to step depolarization positive to –20 mV (Fig. 7a). The rapid inward current was characterized by: reversal potential > 48 mV, activation threshold of ~ −20 mV and maximum amplitude between −20 and 0 mV (Additional file 1: Table S1). This current could be completely inhibited by 300 nM tetrodotoxin (TTX, not shown) and thus these results are consistent with TTX-sensitive voltage gated sodium channels (VGSC) mediating the observed inward currents. The slow rising outward current, likely due to activation of delayed rectifier potassium channels, was inconsistent and thus was not further analyzed.

Naïve SH-SY5Y (Ctr) cells displayed relatively small inward currents when compared to RA differentiated cells (Fig. 7a). Treatment with RA resulted in robust inward sodium currents capable of supporting action potential firing consistent with neuronal-like functionality (Fig. 7a, inset). Current–voltage (IV) relationships were determined from peak currents elicited by 25 ms step depolarizations from −60 to 60 mV, in 10 mV steps, from a holding potential of −80 mV. A 100 ms pre-pulse to −120 mV was implemented to aid VGSC recovery from inactivation. IV relationships from representative cells from each experimental group are shown in the inset in Fig. 7c.

We compared the effects of the three cytotoxins in the electrophysiological properties of RA differentiated SH-SY5Y cells. Overnight incubation with HMB-isatin, VBL and VCR caused graded effects in the electrophysiological properties of RA differentiated SH-SY5Y cells. The voltage dependent parameters of the observed sodium current were not significantly different between RA and the HMB-Isatin and VCR groups (p = 0.7130 and 0.4758, respectively). When compared to the RA group (V_0.5_: −18.6 ± 0.8 mV), only small depolarizing shifts on the activation midpoint (V_0.5_) were observed in VBL (V_0.5_: −13.3 ± 3.2 mV) treated, and naïve (V_0.5_: −13.8 ± 1.4 mV) cells. The latter also displaying an apparent increase in the slope of the activation curve (Fig. 7 and Additional file 1: Table S1).

The magnitude of the VGSC differed between the experimental groups. This was evidenced by comparing the macroscopic conductance (from peak IV plots) and the current density (measured from the ratio of peak current amplitude observed at 0 mV to the cell membrane capacitance in pA/pF) from each group. RA-differentiated SH-SY5Y exposed to HMB-isatin displayed modestly reduced conductance (G_max_: 55.26 ± 9.3 nS, p = 0.5562) and current density (92.2 ± 6.7 pA/pF, p = 0.0885) in comparison to vehicle treated control cells (61.30 ± 5.4 nS, 111.7 ± 8.0 pA/pF, respectively), however these changes did not reach statistical significance (Fig. 6c, Additional file 1: Table S2). In contrast, the macroscopic conductance of VBL (30.81 ± 8.8 nS) and VCR (25.75 ± 5.1 nS) treated groups, as well as their current density (VBL: 65.1 ± 7.3 pA/pF, and VCR: 58.3 ± 8.2 pA/pF) were significantly reduced and comparable to those of naïve non-RA differentiated SH-SY5Y cells (26.99 ± 1.3nS and 61.8 ± 7.9 pA/pF) (Fig. 7c, Additional file 1: Table S2). A decrease in sodium channel function is invariably correlated with decreased excitability therefore; the results presented here may be related to the level of neurotoxicity of the antimitotic drugs studied.

## Discussion

ALL is the most common childhood malignancy and although high remission rates have been achieved, relapse occurs in ~ 20% of patients. This is predominantly due to the development of MDR to chemotherapy [44]. Despite substantial advancements in ALL treatment, the use of *Vinca* alkaloids, particularly VCR, remains the gold standard of care. However, resistance to these drugs as well as their high potential to cause chemotherapy-induced peripheral neuropathy (CIPN) highlights the need to further develop strategies to treat or prevent ALL relapse. The 5,7-dibromo-*N*-alkylisatins are a diverse and growing class of antimitotic agents that have demonstrated the ability to retain potency against clinically resistant cancer cell lines in vitro and reduce primary tumour growth in vivo [28–30]. The current study expands on our understanding of this ability and shows three *N*-alkylisatins, namely HMB-isatin, I4M-isatin and N2M-isatin, to retain potency against drug resistant ALL cell lines. These *N*-alkylisatins also demonstrate a potential for reduced neurotoxicity compared to current clinically used *Vinca* alkaloids, VCR and VBL, suggesting that with further development, the *N*-alkylisatins could be used in the treatment of ALL with reduced potential for the onset of CIPN.

Our in vitro cytotoxicity data demonstrate that while the CEM-VCR R cell line displayed cross-resistance to an array of drugs, the CEM/2ME2-28.8R line was resistant to 2ME2 only. This confirms previous reports that the mechanisms that drive resistance in these two lines differ. 2ME2 is not considered a substrate for P-gp [17, 45] and consistent with this, the 2ME2-selected cell line did not exhibit cross-resistance to agents that are known P-gp substrates including colchicine, paclitaxel, VBL and VCR [46]. Interestingly, the CEM/2ME2-28.8R cell line contains four class I β-tubulin point mutations, S25N, D197N, A248T and K350N [17]. Of these, the S25N mutation lies within the paclitaxel-binding site, the D197N mutation is not within any known drug-binding site on β-tubulin, while A248T and K350N are located within the colchicine-binding site on β-tubulin. We proposed that this line could serve as a tool to further elucidate *N*-alkylisatin binding to β-tubulin, as any change in the tertiary binding site and/or its immediate environment may subsequently affect drug activity. In this study, cytotoxicity assays revealed that all three *N*-alkylisatins retained potency against the CEM/2ME2-28.8R cell line with IC_50_ values not significantly different to that of the CEM parental control cell line. Our competition binding experiments clearly indicate that *N*-alkylisatins compete for binding with a colchicine analog, strongly supporting their binding to the colchicine site. Therefore, the fact that the compounds retain potency against the 2ME2 (known colchicine site ligand [47]) resistant cell line indicates that residues β248 and β350 are not involved in *N*-alkylisatin binding to the colchicine site. Altogether these data are congruent with the observation of Liaw et al. [17] and indicate that, despite the presence of two point-mutations in the colchicine binding site, CEM/2ME2 R cells are not cross-resistant to colchicine or colchicine binding-site agents.

Multiple factors are thought to contribute to the drug resistant phenotype of the CEM-VCR R line [18, 48]. Point mutations in β-tubulin, altered tubulin polymer levels and overexpression of drug efflux-transporters have all been reported to play a role. In order to confirm P-gp-mediated resistance in this line, CEM-VCR R cells were incubated with the fluorescent P-gp substrate R123 in the absence and presence of CSA. CSA is commonly used for in vitro P-gp inhibition studies due to its specificity for P-gp [26]. In this study, treatment of VCR-resistant cells with CSA inhibited R123 extrusion by 49%. We concluded that P-gp mediated efflux is a major contributor to R123 extrusion from these cells, however additional resistance mechanisms such as the overexpression of multidrug resistance-associated proteins (MRPs) and/or lung resistance protein (LRP) may also be employed. MRPs are reported to perform similar functions to P-gp, extruding a range of toxins from cells, including *Vinca* alkaloids [49], however, unlike P-gp, MRP1 does not confer high levels of resistance to paclitaxel [50, 51]. LRP is a component of a protein complex located in the cytoplasm and at the nuclear pore. Given that nuclear pore complexes are able to mediate bidirectional transport of substances between the cytoplasm and nucleus, it is hypothesized that LRP may alter drug transport between these two cellular compartments and sequester drugs in the cytoplasm [52]. Clinical studies investigating the expression of these proteins in pediatric ALL patients show that MRP1 and LRP mRNA is observed in 35.3% and 41.2% of patients, respectively [53]. Moreover, high protein expression levels of MRPs have been linked to unfavorable clinical outcomes in pediatric patients [54], as well as predictive markers of relapse in adult patients [55]. When the MDR CEM-VCR R cell line was exposed to HMB-isatin, I4M-isatin or N2M-isatin, IC_50_ values were not statistically different to those observed for the parental control CEM cell line. This was in contrast to the dose response profiles observed for the commercial anti-cancer agents and further confirms our findings [30] that this class of molecule is not susceptible to P-gp, and potentially MRP and LRP, mediated transport. The exact mechanism by which the *N*-alkylisatins overcome MDR is yet to be uncovered, but we posit that polypharmacology could play a role [56].

Antimitotic agents work by causing dynamic instability of microtubules and ultimately induce tumour cell death. They also interfere with the function of microtubules in axons which mediate neuronal vesicle transport [57–59]. For this reason microtubule inhibitors often exhibit unwanted side effects including peripheral neuropathies. Measuring changes in neurite outgrowth during differentiation is one of the most widely performed phenotypic assays to assess CIPN since the ability of neuronal cells to project membrane extensions from their cell bodies is closely linked to their network function and cell health [60, 61]. We investigated the potential of our *N*-alkylisatins to induce neurotoxicity by comparing their effect on SH-SY5Y neurite length and branch points to VCR and VBL, cytotoxins which are known to cause significant neuronal damage [62]. Differentiated human neuroblastoma cells were treated with drug concentrations of 1, 10 and 100 nM. Previous in vitro tests have shown that treatment with VCR at these concentrations cause minor, moderate, and major neuropathic effects. In this study, the *Vinca* alkaloids and *N*-alkylisatins displayed a dose-dependent effect towards the SH-SY5Y cells, however neurotoxicity associated with VCR and VBL treatment was significantly more pronounced. *N*-alkylisatins induced less damage to neurites than VCR and VBL, even at 10 × higher concentrations than the *Vinca* alkaloids. It is important to note that while the potency of VCR and VBL is between 2.7–13-fold greater than the *N*-alkylisatins against the chemosensitive CEM cell line, the *N*-alkylisatins were up to 17 × more potent than VCR and 2.3 × more potent than VBL in the CEM-VCR R resistant line, making the concentrations used in the neurotoxicity experiments highly relevant. Of the three *N*-alkylisatins tested, HMB-isatin was the least neurotoxic, and was therefore chosen for further assessment in functional patch-clamp assays.

For neurofunctional experiments, we used differentiated SH-SY5Y cells as our model of neurons as they exhibit voltage-gated sodium channels essential to action potential generation and are therefore crucial for neuronal activity and cell survival. The cytotoxic drugs studied here affected sodium current magnitude with varied potency. Macroscopic conductance and current density are influenced by channel expression at the plasma membrane. Our patch-clamp results revealed that the average conductance and current density of HMB-isatin treated cells were not significantly different to those of RA differentiated control cells, which suggests that aspects of channel trafficking and expression in these two groups are akin. In contrast, the sodium currents of VBL and VCR treated cells were significantly smaller and similar in magnitude to the naïve group.

In our electrophysiological experiments, we observed complete inhibition of SH-SY5Y VGSC currents by 300 nM TTX confirming functional expression of only TTX-sensitive sodium channel isoforms [63]. A detailed molecular identification of the involved channels is beyond the scope of this work. Nevertheless, when applied directly (5 nM), none of the cytotoxic drugs produced acute modulatory effects on VGSC mediated activity in RA differentiated cells (not shown). Therefore, these drugs do not appear to act directly on the effector sodium channels. Changes in sodium channel expression in RA-differentiated SH-SY5Y cells can alter sodium current density, leading to downstream changes in action potential generation. Current clamp experiments to assess excitability of our SH-SY5Y neuronal model qualitatively reflected the differences in sodium channel current magnitude observed (Fig. 7a insets). There, it can be observed that the strength of the stimuli required to elicit APs is inversely proportional to the magnitude of the sodium current. Those groups with larger Na_v_ currents, RA and HMB-isatin, fire action potentials upon current injection. VBL treated cells, with somehow lower Na_v_ current, also fired APs but only upon stronger stimuli (i.e. higher current injection), whereas VCR and naïve cells failed to fire under our experimental conditions. Spontaneous firings or trains of spikes in response to prolonged stimulation were not observed in any condition precluding rigorous quantification of the resulting excitability profiles. Perhaps the absence of (repetitive) firing is indicative of incomplete neuronal maturation and/or compromised cell fitness to support firing. Thus, our functional assessment points towards an inherent detrimental role of these drugs in differentiation and/or function of neuron-like SH-SY5Y cells.

Altogether, morphological and functional indicators from this study suggest that cytotoxic drug treatment affects differentiation towards the neuronal phenotype, as tubulin disrupting drugs, HMB-isatin, VBL and VCR possibly alter channel trafficking and/or expression. Other cytotoxic drugs like platinum compounds, contribute to the development of CIPN by impairing the electrophysiological function of dorsal root ganglion neurons demonstrated by a reduction and/or loss of the sensory action potential in nerve conduction studies [64]. The reduction in sodium conductance observed in our in vitro experiments is consistent with reports of reduced excitability in peripheral motor and sensory neurons in VCR treated patients [65]. Increasing evidence supports the association between pain and ion channel modulation of neuronal activity in the peripheral nervous system. Voltage-gated sodium channels are plasma membrane proteins responsible for the upstroke phase of the action potential in electrically excitable cells. VGSC function in macromolecular signalling complexes of a pore-forming α-subunit and associated β-subunits. The β-subunits are members of the immunoglobulin (Ig) domain family of cell-adhesion molecules. They modulate multiple aspects of Na_v_ channel behaviour and play critical roles in controlling neuronal excitability [66]. Defective association between α- and β-subunits has been shown to hinder neurite outgrowth in vitro and in vivo [67]. Furthermore, various studies suggest that clustering of VGSC is regulated by electrical activity and requires an intact actin cytoskeleton [68, 69]. Our findings provide evidence of modulation of sodium conductance whereby its dysfunction may likely contribute to neuropathy and/or neuronal death.

RA treatment significantly increased the density of the voltage-activated Na^+^ channels. The appearance of large inward TTX-sensitive Na_v_ currents is an electrophysiological marker of neuronal-functionality. As a consequence, RA cells became excitable and thus fired action potentials in response to current injection (Fig. 7a, insets). We treated differentiated SH-SY5Y cells with cytotoxic drugs and upon exploration of the electrophysiological properties of their endogenous currents observed evidence supporting differential cytotoxic effect from the drugs studied. Altogether, these data highlight the usefulness of this model to evaluate the functional impact of anticancer drugs on neuronal morphology and cellular excitability using an automated patch clamp platform. In vitro cell based models continue to be indispensable tools in assessing CIPN, especially when screening large numbers of chemical compounds. Although beyond the scope of this manuscript, we acknowledge that further exploration of the mechanisms behind the phenomena are necessary. For example, studies could be conducted in rodent models where multiple behavioural, electrophysiological and pathologic measures can be utilised to evaluate neuropathy and gain further insight into the potential neurotoxicity of *N*-alkylisatins within a whole living organism [27].

## Conclusions

The development of *N*-alkylisatin-based compounds with increased anti-cancer activity is ongoing and advancements in the field could lead to novel molecules that possess superior efficacy towards MDR cancers with lessened neurotoxicity profiles. A current opinion on how to best treat ALL relapse is to prevent it, which makes incorporation of these novel drugs into frontline therapy the most practical approach. Combining newer agents with conventional treatment approaches will likely overcome the challenge of balancing efficacy with toxicity and prevent the development of resistant ALL subclones. Altogether, the results obtained from this study reinforce the potential of *N*-alkylisatins to be considered as candidate drugs for the treatment of patients with relapsed and refractory ALL.

## **Supplementary information**

**Additional file 1: Figure S1.** Growth properties of CEM, CEM-VCR R and CEM/2ME2-28.8R cell lines. **Figure S2.** SH-SY5Y cell viability after treatment with HMB-isatin, VCR and VBL**. Figure S3.** Dose dependent effect of HMB-isatin and VCR on RA-differentiated SH-SY5Y neurite morphology. **Table S1.** Electrophysiological parameters of endogenous sodium currents. **Table S2.** Current density estimates from 3 independent experiments.

## Data Availability

The data that support the findings of this study are available from the corresponding author upon reasonable request.

## Abbreviations

2ME2: 2-Methoxyestradiol
ABC: ATP-binding cassette
ALL: Acute lymphoblastic leukemia
BCRP: Breast cancer resistance protein
CEM/2ME2-28.8R: 2-Methoxyestradiol-resistant CEM cell line
CEM: T-cell acute lymphoblastic leukemia cell line
CEM-VCR R: Vincristine-resistant CEM cell line
CIPN: Chemotherapy-induced peripheral neuropathy
CSA: Cyclosporine A
DMSO: Dimethyl sulfoxide
HMB-isatin: 5,7-dibromo-*N*-(*p*-hydroxymethylbenzyl)isatin
I4M-isatin: 5,7-dibromo-*N*-(1*H*-indol-4-ylmethyl)isatin
LRP: Lung-resistance protein
MDR: Multi-drug resistance
MES-SA: Uterine sarcoma cell line
MRP: Multidrug resistance-associated protein
MRP1: Multi-drug resistance-associated protein 1
MTC: 2-Methoxy-5-(2,3,4-trimethoxyphenyl)-2,4,5-cycloheptatrien-1-one
N2M-isatin: 5,7-dibromo-*N*-(naphthalene-2-ylmethyl)isatin
OD: Optical density
P-gp: P-glycoprotein
R123: Rhodamine 123
RA: Retinoic acid
RI: Resistance index
SH-SY5Y: Human neuroblastoma cell line
T-ALL: T-cell acute lymphoblastic leukemia
TTX: Tetrodotoxin
U937: Monocyte-like histiocytic lymphoma cell line
VBL: Vinblastine
VCR: vincristine
VGSC: Voltage gated sodium channels
Vh: Holding potential
VIPN: Vincristine-induced peripheral neuropathy
