# Supplementary material for: N-alkylisatin-based microtubule destabilizers bind to the colchicine site on tubulin and retain efficacy in drug resistant acute lymphoblastic leukemia cell lines with less in vitro neurotoxicity
Source: Cancer Cell Int. 2020 May 15;20:170. doi: 10.1186/s12935-020-01251-6 (PMC7229617; doi:10.1186/s12935-020-01251-6)
Supplement: Supplementary file 1 — Additional file 1: Figure S1. Growth properties of CEM, CEM-VCR R and CEM/2ME2-28.8R cell lines. Figure S2. SH-SY5Y cell viability after treatment with HMB-isatin, VCR and VBL. Figure S3. Dose dependent effect of HMB-isatin and VCR on RA-differentiated SH-SY5Y neurite morphology. Table S1. Electrophysiological parameters of endogenous sodium currents. Table S2. Current density estimates from 3 independent experiments. [file 12935_2020_1251_MOESM1_ESM.docx]

***N*-alkylisatin-based microtubule destabilizers bind to the colchicine site on tubulin and retain efficacy in drug resistant acute lymphoblastic leukemia cell lines with less *in vitro* neurotoxicity.**

Bryce Keenan, Rocio K. Finol-Urdaneta, Ashleigh Hope, John B. Bremner, Maria Kavallaris, Daniel Lucena-Agell, María Ángela Oliva, Jose Fernando Díaz, and Kara L. Vine

| **Contents** | **Page** |
| --- | --- |
| **Figure S1:** Growth properties of CEM, CEM-VCR R and CEM/2ME2-28.8R cell lines  **Figure S2**: SH-SY5Y cell viability after treatment with HMB-isatin, VCR and VBL  **Figure S3:** Dose dependent effect of HMB-isatin and VCR on RA-differentiated SH-SY5Y neurite morphology | **S1**  **S2**  **S2** |
| **Table S1**: Electrophysiological parameters of endogenous sodium currents | **S3** |
| **Table S2**: Current density estimates from 3 independent experiments | **S3** |

**Figure S1**: Growth properties of parental (CEM; green) and resistant (CEM-VBR R; red and CEM/2ME2-28.8R; blue) ALL cell lines. Cells were seeded at 10,000 cells/well in 96-well microplates and imaged in the IncuCyte ZOOM over 72 h. Values are the mean of triplicates (± SD) and expressed as Change in Confluency (percent increase from time zero). P<0.0001 Two-way ANOVA, Tukey's multiple comparisons test.

**Figure S2**: No significant difference in SH-SY5Y cell viability was detected after treatment with HMB-isatin, VCR and VBL compared to vehicle-treated (control) cells as determined by ordinary one-way ANOVA. ns = not significant.


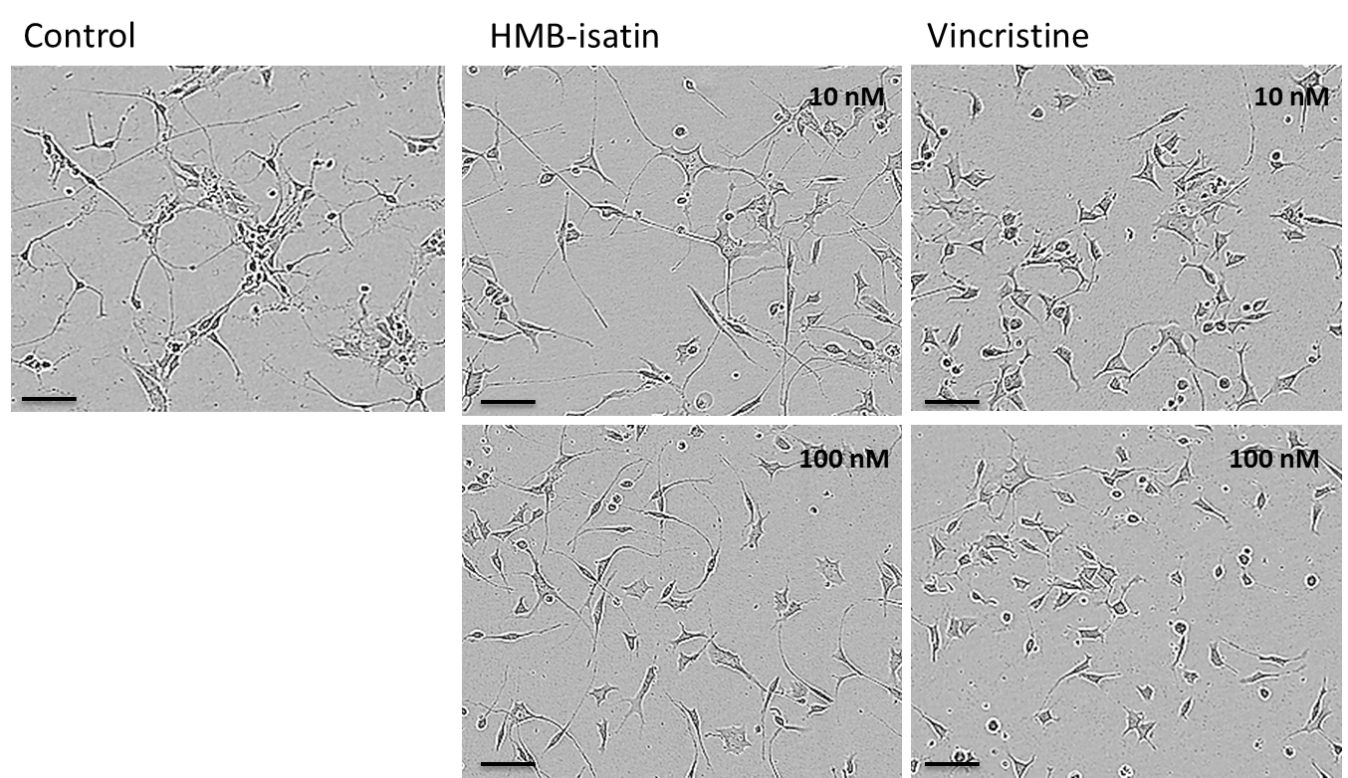


**Figure S3**: Dose dependent effect of HMB-isatin and VCR on RA-differentiated SH-SY5Y neurite morphology after 48 h. Scale bar represents 100 μm.

**Table S1. Electrophysiological parameters of endogenous sodium currents.**

|  | V_0.5_ (mV) | | | Slope | | | G_max_ (nS) | | | V_rev_ (mV) | | |
| --- | --- | --- | --- | --- | --- | --- | --- | --- | --- | --- | --- | --- |
|  | mean±sem | n | p | mean±sem | n | p | mean±sem | n | p | mean±sem | n | p |
| Ctr | -13.8±1.4 | 12 | 0.0043 | 5.9±0.6 | 12 | 0.0202 | 26.99±1.3 | 7 | 0.0001 | 48.5±2.1 | 7 | n.s. |
| RA | -18.6±0.8 | 24 |  | 4.5±0.3 | 24 |  | 61.30±5.4 | 8 |  | 51.5±1.9 | 8 | n.s. |
| Isa | -17.9±1.2 | 11 | 0.7130 | 4.5±0.3 | 11 | 0.9133 | 55.26±9.3 | 6 | 0.5662 | 48.2±2.8 | 6 | n.s. |
| VBL | -13.3±3.2 | 5 | 0.0333 | 4.5±0.3 | 5 | 0.9615 | 30.81±8.8 | 5 | 0.0094 | 48.3±4.6 | 5 | n.s. |
| VCR | -17.4±1.3 | 11 | 0.4758 | 4.5±0.3 | 11 | 0.9740 | 25.75±5.1 | 5 | 0.0010 | 49.1±2.1 | 5 | n.s. |

**Table S2. Current density estimates from 3 independent experiments.**

|  | Exp I | | |  | Exp II | | |  | Exp III | | |
| --- | --- | --- | --- | --- | --- | --- | --- | --- | --- | --- | --- |
|  | Current density  (pA/pF) | | |  | Current density  (pA/pF) | | |  | Current density  (pA/pF) | | |
|  | mean±sem | n | p |  | mean±sem | n | p |  | mean±sem | n | p |
| Ctr | 61.8±7.9 | 7 | 0.0007 |  | 66.2±7.4 | 8 | 0.0001 |  | -69.9±3.4 | 7 | 0.0002 |
| RA | 111.7±8.0 | 8 |  |  | 143.1±11.6 | 8 |  |  | -100.5±4.8 | 8 |  |
| Isa | 92.2±6.7 | 7 | 0.0885 |  | 112.9±5.0 | 6 | 0.0544 |  | -90.9±3.9 | 7 | 0.1636 |
| VBL | 65.1±7.3 | 5 | 0.0022 |  | 78.6±5.0 | 8 | 0.0002 |  | -75.3±5.8 | 7 | 0.0051 |
| VCR | 58.3±8.2 | 8 | 0.0004 |  | 79.1±7.8 | 8 | 0.0004 |  | -71.6±5.4 | 5 | 0.0026 |
